# Physician burnout in the context of the COVID-19 pandemic: prevalence and associated factors among resident doctors and consultants in Delta State, Nigeria

**DOI:** 10.1371/journal.pmen.0000175

**Published:** 2025-05-07

**Authors:** Nnamdi Stephen Moeteke, Ezinneamaka Erhirhie

**Affiliations:** 1 Department of Community Medicine, Delta State University Teaching Hospital, Oghara, Nigeria; 2 Department of Community and Public Health, Idaho State University, Pocatello, Idaho, United States of America; 3 Department of Family Medicine, Delta State University Teaching Hospital, Oghara, Nigeria; 4 Mersey and West Lancashire Teaching Hospitals NHS Trust, Merseyside, United Kingdom; PLOS: Public Library of Science, UNITED KINGDOM OF GREAT BRITAIN AND NORTHERN IRELAND

## Abstract

Burnout is a state of emotional, physical, and mental exhaustion caused by long-standing, poorly managed workplace stress. Resident doctors (RDs) and consultants provide specialized medical care, and the strain of their job predisposes them to the three domains of burnout: Emotional Exhaustion (EE), Depersonalization (DP), and diminished Personal Accomplishment (PA). Globally, this public health crisis worsened with the overwhelming effect of COVID-19 on health systems. This study assessed the prevalence and associated factors of burnout among RDs and Consultants in tertiary hospitals in Delta State, Nigeria, during the pandemic. A cross-sectional design was employed. Previously validated instruments (the Maslach Burnout Toolkit, and the Pandemic Experience and Perception Survey) were used to collect data via an online survey. The questionnaire was sent to physicians selected by a multistage sampling. The proportion of participants with a high grade in each of the domains of burnout was obtained. Stepwise analyses from bivariate to multivariate were done to obtain adjusted odds ratios. The prevalence of high-grade burnout in EE, DP, and PA was 35.1%, 13.2%, and 33.3% respectively. Relative to those ≤ 30 years, the age group 41 – 50 years had less likelihood of high EE (AOR 0.050; 95% CI 0.004 – 0.651). Other independent predictors of EE were manageable workload (AOR 0.094; 95% CI 0.027 – 0.328), reward for work (AOR 0.427; 95% CI 0.205 – 0.892), and good leadership (AOR 0.525; 95% CI 0.113 – 0.929). This study suggests that the determinants of burnout among RDs and consultants are mainly contextual factors in the work setting. Future interventions to control physician burnout in Nigerian healthcare systems should be geared towards promoting an institutional culture of leadership, manageable workloads, and appropriate rewards for good performance.

## Introduction

Medical practice and its demands of saving lives can be psychologically stressful [1]. Long on-the-job hours, including call duties, are the norm[2]. These strains are amplified in developing countries like Nigeria, with constant organizational challenges and resource shortages, which lead to incessant brain drain and more workload for the remaining manpower[2,3]. These predispose physicians and other healthcare professionals to burnout. Burnout is characterized by a combination of associated symptoms emanating from long-standing poorly managed workplace stress, with three facets, namely:

a) Emotional exhaustion (EE)—feelings of being drained or energy-depleted
b) Depersonalization (DP)—increased mental and emotional distance from one’s job, or feelings of negativism and cynicism towards one’s job
c) Diminished personal accomplishment (PA)—reduced professional self-esteem or feelings of low success [4].

Globally, there was an increase in burnout among health professionals during the COVID-19 pandemic, with many having to work in overwhelmed health systems, associated with high levels of risk, anxiety, and mortality[5]. The impact of burnout on physicians includes depression and poor general health, individual and workplace impairment, substance abuse, and suicide. These ultimately affect the quality of care of patients (through decreased professional capacity, indifference and loss of empathy for patients, and higher chances of healthcare errors), the health organizations/systems (absenteeism and high turnover of already trained personnel), and colleagues/families of affected doctors (relationship friction), making it a ‘public health crisis’ [6–9]. A survey conducted in Eastern Nigeria just before the pandemic got to the country revealed that the prevalence of burnout was 69% among healthcare workers, with as high as 40% screening positively for depression, and the domains of burnout correlating strongly with decreased productivity[10]. Therefore, the pressure on Nigerian healthcare systems due to COVID-19 exacerbated the challenges already faced by physicians in the country. The latest ‘Physicians’ Oath’ acknowledges the welfare of the medical doctor, and inductees are now required to swear to “attend to my own health, well-being, and abilities in order to provide care of the highest standard”[11]. However, unlike somatic ailments, smoldering symptoms of burnout are often put on the back burner, especially in developing countries where the condition is under-reported[12]. Though the term “burnout” has been around since the 1970s, it is acknowledged that more data is continually required to design evidence-based control measures among physicians[1].

The Maslach Burnout Inventory (MBI), a 22-item questionnaire, is the gold standard and the most prominent tool for measuring burnout as defined by the World Health Organization (WHO) [9,13]. It has been validated by countless research on burnout that have been done since the tool was developed over four decades ago[14]. MBI measures burnout in each of the three dimensions using questions with responses on a 7-point scale: ‘Never’, ‘a few times a year’, ‘once a month’, ‘a few times per month’, ‘once a week’, ‘a few times a week’, and ‘every day,’ with the scores ranging from 0 - 6. There are nine questions for ‘Emotional Exhaustion’ (EE), five for ‘Depersonalization’ (DP), and eight for ‘Personal Accomplishment’(PA) [15]. In the original MBI Manual [16], scores in each domain are categorized into “low,” “moderate,” and “high” burnout (Table 1). The three dimensions are non-cumulative.

**Table 1:** Categorization of MBI Scores [16].

Subscale	Category	Cut-off Scores
**Emotional exhaustion** **(Score: 0 – 54)**	High	≥ 27
	Moderate	19 – 26
	Low	0 – 18
		
**Depersonalization** **(Score: 0 – 30)**	High	≥ 10
	Moderate	6 - 9
	Low	0 - 5
		
**Diminished personal accomplishment** **(Score: 0 – 48)**	High	0 - 33
	Moderate	34 - 39
	Low	≥40

The absence of a universal interpretation of the MBI whereby overall burnout is presented as a single binary variable is problematic. While some investigators describe overall burnout as the presence of high burnout in all three domains, others have considered study participants to be truly burnt out if at least two sub-scales are in the ‘high’ category [17,18]. Other authors see burnout as having either high emotional exhaustion or high depersonalization [19]. Overall burnout has also been estimated based on participants having a high burnout in at least one of the three domains[15] These variations make it difficult to compare studies, with some studies whose authors belong to the first two schools of thought profoundly underestimating rates, at least potentially[20]. While the developer of the instrument supports the idea of defining clinical burnout as the presence of a high EE burnout alongside either a high DP or PA burnout, she recommends that when assessing for associations between burnout and other variables, it is best to use individual subscale scores as numerical data, or, less preferably, to report burnout in each domain as a categorical variable based on the already established cut-offs for ‘high’, ‘moderate’, and ‘low’ [20].

In Nigeria, resident doctors (RDs) are registered medical/dental graduates working and undergoing specialized training (under the supervision of consultants) in health facilities (mostly tertiary hospitals) accredited by the regulatory bodies for graduate medical education[21]. RDs begin their training as Registrars, and are promoted to the rank of Senior Registrar when they pass the Part 1 Fellowship Examination for their specialty which is usually taken after a minimum of three years. A consultant is a medical/dental practitioner who, having completed the residency training and passed the Part 2 Fellowship Examination, works as a specialist in a health facility or medical school [22]. There are about 17,000 RDs—about 40% of the doctor population—and over 4000 consultants in Nigeria[23,24]. It is difficult to ascertain the prevalence of overall burnout among physicians in low- and low-middle-income countries due to variations in research instruments and criteria. Nine out of the studies obtained from literature search employed the MBI and the true prevalence of high EE, DP, and PA burnout may only be estimated from these studies. The papers that examined both RDs and consultants, like this current study, reported levels of high EE, DP, and PA burnout ranging from 20% - 39.4%, 30% - 71%, and 25% - 50.3%, respectively [25–28].

The relationship between socio-demographic characteristics and physician burnout is unclear as the evidence varies[15,25,29–31]. However, the higher likelihood of burnout among younger physicians and those with fewer years in practice has been highlighted[25,26,29,32]. More consistent predictors of physician burnout are work- and training-related factors such as frequency/duration of being on call, number of work hours/workload, remuneration/economic problems, organizational support, recognition from hospital managers, getting adequate skill development and professional training including pandemic-related training, satisfaction with hospital infection control measures, and frequency in dealing with suspected, confirmed, and critical cases of COVID-19. These indicate that determinants of burnout are mostly contextual factors related to organizational arrangements and management systems in the work setting [33]. This is corroborated by the qualitative aspect of the study by Ghazanfar et al [34]. In low- and low-middle-income countries, only five studies done in Egypt (2), Ethiopia, Pakistan, and Sri Lanka explored, apart from workload, different aspects of these institutional predictors, albeit superficially[25,30,32,34,35]. Furthermore, only two studies done in Egypt provide data on the prevalence and associated factors of physician burnout during the COVID-19 pandemic[25,32]. There is a paucity of studies in Nigeria involving both residents and consultants, assessing organizational factors and providing data on physician burnout in the context of the pandemic. This study sought to fill the gap in the literature as regards physician burnout during the COVID-19 pandemic and highlight critical areas of strength and or weakness in healthcare organizations to guide prevention and control strategies. Therefore, the aim of this study was to assess the prevalence of burnout among RDs and Consultants working in tertiary health facilities in Delta State, Nigeria, and determine the associated factors such as socio-demographic characteristics, features of the work setting, and experiences/perceptions of work during the pandemic.

## Methods and materials

### Ethics statement

Ethical clearance was obtained from the Health Research Ethics Committee of Delta State University Teaching Hospital (Approval Number HREC/PAN/2021/037/0426). The licenses to use the Maslach Burnout Toolkit and the Pandemic Experience and Perceptions Survey (PEPS) were purchased from the copyright holder, Mind Garden, Inc. The study participants had to go through the Participant Information Sheet (PIS) and consent statements after clicking on the link to the online survey. They were informed that they could withdraw from the study without repercussions and were required to tick checkboxes indicating their consent before they could proceed to answer the questionnaire. The PIS contained the researchers’ telephone numbers and email addresses to enable potential participants to ask questions if needed. It also had contact details of support groups/organizations that participants could contact if the questionnaire caused emotional distress. There were no identifiers on the questionnaire, and the online survey had no IP links to ensure anonymity. The data was stored in a password-protected laptop that was accessible to the investigators only. There was no incentive or compensation for participants.

### Study design

A cross-sectional design, which gives a ‘snapshot’ of an outcome of interest in a population, was used. This was most appropriate since it is the only design that can both measure the health status of a given population at a specific place and time and determine associated factors for the outcome of interest [36].

### Study setting

This study was done in the two tertiary health facilities in Delta State, Nigeria. These are government-owned hospitals providing the highest level of specialized and critical healthcare to inhabitants of the state and its environs. Each hospital provides services in these specialties: anesthesia, community medicine, emergency medicine, family medicine, internal medicine, obstetrics & gynecology, ophthalmology, orthopedics, otorhinolaryngology, pediatrics, radiology, and the various subspecialties of surgery. There were a total of 502 RDs and Consultants working in both institutions.

The index case of COVID-19 in Nigeria was reported on February 27, 2020, while the first case in Delta State was confirmed on April 7, 2020[37]. The first wave of the pandemic in the country lasted from February to October 2020; the second wave from October 2020 to June 2021; and the third wave from June to November 2021. The fourth wave began in December 2021 and started to flatten in January 2022. The second wave was the largest, with the number of new cases per month reaching a record high of 43,635 in January 2021. Though the fourth wave had the shortest duration, it was also remarkable as new cases increased by 500% in two weeks, and the week of December 20 – 26 witnessed an unprecedented 14,078 new cases per week[38–45].

### Selection criteria

RDs and consultants who had worked in the facilities for ≥ 1 year were included. Supernumerary RDs and consultants hired on temporary or part-time contracts were excluded.

### Sample size calculation and sampling

The formula for obtaining the minimum sample size (n) for identifying a proportion in a population was used:


$$n\;=\;1.96^{2}\;P(100-P)/E^{2}$$

where 

n = minimum sample size

P = proportion of the population with burnout = 75.5%, from a previous study [31]

E = acceptable margin of error (5%)


$$n\;=\;1.96^{2}\;x\;0.755\;x\;0.255/0.05^{2}\;\approx \;296$$

Since the total number of RDs/Consultants is less than 10,000, the following correction formula was used to obtain a corrected sample size, n_c._


$$\frac{n\;Pt}{n+(Pt-1)}$$

Where n is the uncorrected sample size, and Pt is the total population of RDs/consultants.


nc = (296 × 502)/ (296 + [502 – 1]) ≈ 187

Assuming a non-response rate of 10%, this required sample size was increased by 10% to 206.

The lists of RDs and consultants for both hospitals (with distribution by specialty) were obtained. Based on the total number of RDs and consultants in each hospital, the total number in each specialty and the numbers of RDs and consultants in each specialty, 206 RDs/consultants were selected by multistage sampling technique. This involved stratified sampling in three stages to proportionately allocate participants to the two hospitals, to individual specialties in each hospital, and to the three cadres (registrar, senior registrar, and consultant) in each specialty within each hospital. Participants were then selected by simple random sampling technique (balloting method) for individual cadres in each specialty for the two hospitals.

### Recruitment

The directory of the physicians’ association in the state (to which these researchers belong), which is readily available to members, was used. All doctors have access to this document as a shared resource, and these researchers are permitted to send recruitment invites out to the list with no need for a gatekeeper. The study was advertised in the doctors’ WhatsApp groups with a brief description of the purpose, aim/objectives, eligibility criteria, and contact details of the researcher. The potential participants were given the opportunity to contact the researchers if they had any questions and had two weeks to consider their participation. Thereafter, the web link to the online participant information sheet (PIS), consent form, and questionnaire was sent via WhatsApp to the selected individuals, with a friendly reminder after two, four, and six weeks, respectively. Data collection was done from January 13 to March 5, 2022.

### Method of data collection

Transform Survey Hosting [46] was used. The questionnaire (about 25 minutes to complete) consisted of four sections: socio-demographic/personal professional data, the Maslach Burnout Toolkit, and the Pandemic Experience and Perceptions Survey (PEPS). Maslach Burnout Toolkit “combines the Maslach Burnout Inventory (MBI) and the Areas of Worklife Survey (AWS) to measure burnout in the Worklife context” [47].

The AWS, which has proven validity and reliability in different occupational settings, is a companion tool to the MBI and assesses workplace characteristics that could predict the occurrence of burnout[13]. AWS has 28 items (scored on a five-point Likert scale) which assess the perception of ‘Workload’ (5 items), ‘Control’ (4), ‘Reward’ (9), ‘Fairness’ (6), and ‘Organizational Values’ (4). The average score for each sub-component was obtained.

The PEPS (35 items scored on a five-point Likert scale) is a powerful tool for assessing employees’ experiences and perceptions during a pandemic. The question items are in five categories: ‘Extent of Impact’ (3 items), ‘Adequacy of Resources’ (5), ‘Risk Perception’ (7), ‘Worklife’ (7), and ‘Leadership’ (10) [48]. The total score for each category was obtained.

The survey instruments were pretested among 11 RDs and 10 consultants of a tertiary hospital in a neighboring state to ensure suitability for the study population, and they were revised for clarity. The PEPS had to be adapted for this study by slightly modifying the questions to make them specific to the current pandemic. For example, under ‘Leadership’, the question “How did you experience leadership during the pandemic period” was changed to “How do you experience leadership during the ongoing COVID-19 pandemic.” Similarly, among the items for ‘Risk Perception’, “For the ongoing COVID-19 pandemic, how much risk to your family do you perceive” was used instead of “For the relevant period, how much risk to your family did you perceive.”

### Description of the dependent variables

The dependent variables were EE, DP and PA. Burnout was indicated by high category, i.e., EE score ≥ 27, or DP score ≥ 10, or PA score ≤ 33 [9]. Each participant was allocated to ‘burnout’ or ‘no burn out’ to create a dichotomous dependent variable for each subscale. The prevalence of burnout was measured by the proportion (percentage) of participants who met the cut-off in at least one subscale.

### Data analyses

The dataset was checked for missing data and there was none for all the listed variables. The Chi-squared test was used to determine if there was an association between categorical independent variables (e.g., age group, marital status) with each domain of burnout. For continuous independent variables (e.g., score for perception of risk), association with scores for each domain of burnout was tested using Pearson correlation. To identify independent predictors of each domain of burnout, all categorical and continuous variables that were statistically significant on bivariate analyses were introduced into a binary logistic regression model. The level of statistical significance (α) was set at 0.05 for all analyses.

## Results

Only 114 people submitted the filled questionnaire, giving a response rate of 55.3%. The average age of respondents was 40 years (Table 2). Of the 114 respondents, 40 (35.1%) had high EE, 15 (13.2%) had high DP, and 38 (33.3%) had high PA burnout. There was high burnout in at least one domain in 46 respondents (40.4%) (Table 3).

**Table 2:** Socio-demographic and professional characteristics of respondents.

Characteristic of Respondents		Total, N = 114	
		*n*	%
Age (years)	≤30	10	8.8
	31 – 40	55	48.2
	41 – 50	42	36.8
	≥ 51	7	6.1
	Mean ± SD	40.30 ± 6.27	
			
Sex	Male	66	57.9
	Female	48	42.1
			
Marital status	Single	11	9.6
	Married	96	84.2
	Separated	3	2.6
	Divorced	2	1.8
	Widow	2	1.8
			
Specialty category	Medical-related	64	56.6
	Surgical-related	41	36.3
	Diagnostic	8	7.1
			
Rank	Consultant	22	19.3
	Senior resident doctor	52	45.6
	Junior resident doctor	40	35.1
			
Number of years post- graduation from medical school	≤10 years	40	35.1
	11 – 20 years	58	50.9
	21 – 30 years	15	13.2
	≥ 31 years	1	0.9

**Table 3:** Distribution of burnout categories among respondents.

Burnout domain		Frequency (%)
**Emotional Exhaustion (EE)**	Low burnout	53 (46.5%)
	Moderate burnout	21 (18.4%)
	High burnout	40 (35.1%)
		
**Depersonalization (DP)**	Low burnout	76 (66.7%)
	Moderate burnout	23 (20.1%)
	High burnout	15 (13.2%)
		
**Personal Accomplishment (PA)**	Low burnout	45 (39.5%)
	Moderate burnout	31 (27.2%)
	High burnout	38 (33.3%)
		
**Overall Burnout (presence of at least one of High EE, High DP, and High PA burnout**	Yes	46 (40.4%)
	No	68 (59.6%)

The categorical variables significantly associated with EE were age group and number of years post-graduation (Table 4). Only age group was significantly associated with DP (Table 5). EE scores had a weak positive correlation with number of work hours per week, and scores for perception of risk; a weak negative correlation with scores for sense of control, community, fairness, values, and adequacy of resources; and a moderate negative correlation with scores for manageability of workload, reward, and experience of leadership. A similar pattern was obtained for DP scores except that all the correlations were weak (Table 6).

**Table 4:** Association between Emotional Exhaustion (EE) and categorical independent variables.

Characteristic of Respondents		Emotional Exhaustion (*N* = 114)						χ^2^	*P*-value
		Low		Moderate		High			
		n	%	n	%	n	%		
Age (years)	≤30	2	3.8	0	0.0	8	20.0	18.131	^*^0.006
	31 – 40	23	43.4	10	47.6	22	55.0		
	41 – 50	22	41.5	11	52.4	9	22.5		
	≥ 51	6	11.3	0	0.0	1	2.5		
									
Sex	Male	27	50.9	13	61.9	26	65.0	2.018	0.365
	Female	26	49.1	8	38.1	14	35.0		
									
Marital status	Single	4	7.5	1	4.8	6	15.0	5.243	0.731
	Married	45	84.9	19	90.4	32	80.0		
	Separated	2	3.8	0	0.0	1	2.5		
	Divorced	1	1.9	1	4.8	0	0.0		
	Widow	1	1.9	0	0.0	1	2.5		
									
Specialty category	Medical	33	63.4	10	47.7	21	52.5	7.734	0.102
	Surgical	16	30.8	7	33.3	18	45.0		
	Diagnostic	3	5.8	4	19.0	1	2.5		
									
Rank	Consultant	14	26.4	3	14.3	5	12.5	8.415	0.078
	Senior Registrar	24	45.3	13	61.9	15	37.5		
	Registrar	15	28.3	5	23.8	20	50.0		
									
Number of years post- graduation from medical school	≤10 years	16	30.2	4	19.0	20	50.0	15.602	^*^0.016
	11 – 20 years	24	45.3	16	76.2	18	45.0		
	21 – 30 years	12	22.6	1	4.8	2	5.0		
	≥ 31 years	1	1.9	0	0.0	0	0.0		

**χ**^**2**^ **=** Chi-squared test statistic |

* statistically significant

**Table 5:** Association between Depersonalization (DP) and categorical independent variables.

Characteristic of Respondents		Depersonalization (*N* = 114)						χ^2^	*P*-value
		Low		Moderate		High			
		n	%	n	%	n	%		
Age (years)	≤30	2	2.6	3	13.0	5	33.3	16.482	^*^0.011
	31 – 40	37	48.7	12	52.2	6	40.0		
	41 – 50	32	42.1	7	30.4	3	20.0		
	≥ 51	5	6.6	1	4.3	1	6.7		
									
Sex	Male	44	57.9	14	60.9	8	53.3	0.212	0.900
	Female	32	42.1	9	39.1	7	46.7		
									
Marital status	Single	5	6.6	3	20.0	3	20.0	10.190	0.252
	Married	67	88.2	19	66.7	10	66.7		
	Separated	2	2.6	0	0.0	1	6.7		
	Divorced	2	2.6	0	0.0	0	0.0		
	Widow	0	0.0	1	4.3	1	6.7		
									
Specialty category	Medical	45	60.0	12	52.2	7	46.7	1.277	0.865
	Surgical	25	33.3	9	39.1	7	46.7		
	Diagnostic	5	6.7	2	8.7	1	6.6		
									
Rank	Consultant	16	21.1	2	8.7	4	26.7	8.296	0.081
	Senior Registrars	39	51.3	10	43.5	3	20.0		
	Registrars	21	27.6	11	47.8	8	53.3		
									
Number of years post- graduation from medical school	≤10 years	22	28.9	9	39.2	9	60.0	9.310	0.157
	11 – 20 years	42	55.3	13	56.5	3	20.0		
	21 – 30 years	11	14.5	1	4.3	3	20.0		
	≥ 31 years	1	1.3	0	0.0	0	0.0		

**χ**^**2**^ **=** Chi-squared test statistic |

* statistically significant

**Table 6:** Correlation between burnout scores and continuous independent variables.

Characteristic of Respondents		Emotional Exhaustion	Depersonalization	Diminished Personal Accomplishment
Work hours per week	***r***	0.231	0.186	0.057
	***p*-value**	^*^0.014	^*^0.047	0.547
				
Number of calls per month	***r***	0.017	0.153	-0.101
	***p*-value**	0.856	0.105	0.286
				
Manageability of workload^#^	***r***	-0.538	-0.269	-0.129
	***p*-value**	^*^ < 0.0001	^*^0.004	0.173
				
Control over work life^#^	***r***	-0.309	-0.278	-0.023
	***p*-value**	^*^0.001	^*^0.003	0.808
				
Reward for work^#^	***r***	-0.411	-0.165	-0.021
	***p*-value**	^*^ < 0.0001	0.080	0.822
				
Community at workplace^#^	***r***	-0.280	-0.180	0.013
	***p*-value**	^*^0.003	0.055	0.890
				
Fairness at workplace^#^	***r***	-0.326	-0.225	0.070
	***p*-value**	^*^ < 0.0001	^*^0.016	0.459
				
Workplace values^#^	***r***	-0.307	-0.252	0.008
	***p*-value**	^*^0.001	^*^0.007	0.936
				
Impact of pandemic^#^	***r***	0.107	0.011	0.037
	***p*-value**	0.256	0.910	0.696
				
Adequacy of resources^#^	***r***	-0.263	-0.175	0.010
	***p*-value**	^*^0.005	0.063	0.912
				
Perception of risk	***r***	0.241	0.237	-0.001
	***p*-value**	^*^0.010	^*^0.011	0.989
				
Leadership	***r***	-0.452	-0.308	0.030
	***p*-value**	^*^ < 0.0001	^*^0.001	0.749

# Perception and experience of Respondents | *r* = Pearson correlation coefficient |

* Statistically significant

The multivariate model for high EE showed decreasing likelihood of high EE with older age groups, though this was statistically significant only for those aged 41–50 years who had a 95% decrease in odds compared to the group of ≤ 30 years. The AWS and PEPS variables which were independent predictors of high EE were ‘manageability of workload’, ‘reward for work’, and ‘experience of leadership’ (Table 7). There was no statistically significant association between DP and all the predictors entered into the model.

**Table 7:** Result of binary logistic regression model for factors associated with high Emotional Exhaustion.

Characteristic of respondents		Adjusted odds ratio	95% CI		*P*-value
			Lower	Upper	
**Categorical variables**	**Age group**				
	≤30 years^				
	31 – 40 years	0.189	0.021	1.688	0.136
	41 – 50 years	0.050	0.004	0.651	^*^0.022
	≥ 51 years	0.008	0.000	1.056	0.053
	**Years post-graduation**				
	≤10 years^				
	11 – 20 years	0.810	0.189	3.469	0.776
	21 – 30 years	3.249	0.146	72.486	0.457
	≥ 31 years	0.000	0.000	0.000	1.000
					
**Continuous variables**	Work hours per week	1.001	.986	1.017	0.862
	Workload^#^	0.094	0.027	0.328	^*^0.000
	Control^#^	2.118	0.838	5.352	0.113
	Reward^#^	0.427	0.205	0.892	^*^0.024
	Community^#^	0.799	0.343	1.861	0.602
	Fairness^#^	0.843	0.266	2.674	0.772
	Workplace values^#^	0.694	0.281	1.715	0.429
	Adequacy of resources^#^	1.018	0.841	1.231	0.856
	Risk perception	1.047	0.936	1.170	0.424
	Leadership^#^	0.525	0.113	0.929	^*^0.043

# Perception and experience of respondents | ^Reference category | CI = Confidence Interval |

* statistically significant

## Discussion

### Implications of the findings

The findings meet the aims of this study, which are to fill the gap in the literature in terms of the prevalence of physician burnout in Nigeria during the pandemic and, beyond socio-demographic characteristics, to explore predictors such as experiences and perceptions of the pandemic and workplace characteristics. To the best of our knowledge, this is the first study to provide data in this regard in Nigeria.

The results indicate that the prevalence of high burnout in the EE (35.1%) and PA (33.3%) domains, as well as overall burnout (40.4%), is quite elevated among Nigerian physicians who provide specialized and critical care for the populace. However, these levels are slightly less than was obtained in Benin City, Nigeria and Pakistan [15,18], and far less than the other studies in South Africa, Ethiopia, Egypt, and Iran [28,32,33,35]. The only study with the closest rates (EE 30%, DP 30%, and PA 25%) was that among oncology RDs and consultants in Egypt [26]. The higher rates in most studies could be because they involved only RDs (who are younger doctors and undergo more work stress), or were focused on one specialty with a greater tendency for burnout, such as emergency medicine.

For the role of socio-demographic factors, there is little discrepancy with other studies that employed multivariate analyses to ascertain independent predictors. This study’s observation of significantly higher burnout amongst younger physicians mirrors the findings of others[26,27,35]. Unlike Fernando and Samaranayake [32], who observed higher risk among females, and Goyal and colleagues [49], who noted a stronger association with male sex, this study found no gender predisposition in keeping with the majority of other studies [15,18,25–28,32,33,35]. The unique cultural and systemic factors in Nigeria (and similar settings) could explain why gender does not emerge as a significant predictor of burnout. Generally, both male and female physicians face enormous societal expectations, although these might differ in form. For instance, while women may be expected to juggle professional and domestic responsibilities (given traditional gender roles around caregiving and homemaking), men may experience the pressure of being the primary earners and decision-makers of their families. However, the distinction in these forms of stress may not be significant enough to differentiate the burnout experiences between the two genders, especially if both groups encounter similar systemic challenges such as high workloads and lack of resources in their professional life. Secondly, though the medical profession in Nigeria continues to be male-dominated[50], the expectations for physicians are the same, regardless of gender. Female physicians, particularly those entering the field in recent decades, are usually expected to adapt to their demanding roles just like their male counterparts.

Unlike in the study by Soltan et al [26], where there was no association between burnout and workload, this study showed manageability of workload as a negative correlate of EE, which is in agreement with most others where excessive perceived stress of work overload, higher occupational stress index, dealing with critical cases, more work hours, seeing more patients, and more frequent calls increased burnout[15,27,28,32,35,49]. Also, the observed negative linear relationship between burnout and reward at work supports other studies that found that burnout has an inverse relationship with recognition from hospital managers, monthly salary [35], celebrating accomplishments, having enough money[18], and absence of economic problems and concerns about future career [28]. The absence of an association with control of work life and community is in line with a study that found that burnout is not affected by lack of control over office/training or poor cordiality with colleagues[18].

Regarding the COVID-19 context, though adequacy of resources and perception of risk showed a relationship with burnout on bivariate analysis, these turned out to be insignificant on logistic regression, like the study by Mahmood et al[18]. This is at variance with the finding elsewhere of an association between burnout and receipt of pandemic-related training, satisfaction with organizational infection control measures, having a colleague who is infected, and fear of the coronavirus infection [25]. The observed protective effect of good leadership is in keeping with other research that highlighted the similar role of a supportive organizational and work environment [18,35].

In general, this study supports the general suggestion in the wider literature that, apart from age, rather than socio-demographic and personal professional variables (such as specialty and years of practice), the determinants of burnout are essentially contextual factors related to organizational arrangements and management systems in the work setting[33]. The observed association between burnout and contextual factors (such as workload, reward, and leadership) contributes to a broader understanding of physician burnout in resource-constrained settings by highlighting how these elements interact to create an environment where burnout is more likely to occur and more difficult to mitigate. In Nigeria, for example, physicians continue to face high patient volumes, insufficient staffing, limited tools, and poor remuneration[51]. It is also acknowledged that healthcare professionals occupying leadership positions in both the public and private sectors often lack the leadership skills and competencies required for their roles[52]. Recognizing these patterns can lead to more effective strategies aimed at supporting physician well-being and improving patient care in such challenging settings.

### Strengths/limitations of the study and discussion of the research process

The strengths of this study include the use of renowned and previously validated instruments which assessed several potential predictors, and multivariate analyses to ascertain their independent association with the outcomes of interest. Only cross-sectional studies were included in the literature review for direct comparability.

None of the significant associations with DP observed on bivariate analysis was maintained after logistic regression. This may have been due to depreciation in degrees of freedom (and, hence, precision) because the variables included in the regression model were too many for the dataset. It has been suggested that the ratio of events to parameters in a regression model should be 10:1[53]. Therefore, with only 15 persons having high DP, having seven variables in the model was likely a pitfall. Another reason for the initially significant predictors becoming insignificant may be having two or more input variables that have a correlation, as is most likely the case for age and number of years post-graduation. The consequence is that the effect of each input variable gets dispersed and attenuated [53]. It could have been better to use only one of the related parameters.

A serious limitation of this study is the low response rate with its implications for the external validity since the sample may be unrepresentative of the study population, as individuals who turn down research invitations tend to differ from those who participate [54]. The extent of this non-response bias could have been determined by comparing the characteristics of responders with the same characteristics of non-responders [55], but it was impracticable to obtain tangible information about non-respondents. The sample size was also below the calculated required minimum, which means that it had a reduced statistical power to detect statistically significant differences where such differences exist, and the survey estimates may lack precision[56]. Survey response rates have been on the decline in recent times due to progressively increasing time pressures on people, and medical doctors respond less than other health workers[56]. This may have affected this study, especially with the shortage of medical personnel due to the massive brain drain and the impact of the COVID-19 pandemic on health personnel and systems. Making repeated contact with selected study subjects is a strategy for improving recruitment [54], and this was employed in this study with three reminder WhatsApp messages (at roughly two-week intervals) after the initial contact. These were done courteously, always bearing in mind respect for autonomy [55]. 

One person replied that he started the survey but could not complete it because the questions triggered him. This suggests that the obtained prevalence of burnout is less than the true picture since non-response, even as low as 25%, distorts estimates of disease burden when the disorder itself is responsible for the non-response [54]. As a duty of care, the physician who experienced emotional distress was directed to organizations where he could get help. Many of the doctors gave strong feedback that the questionnaire was too long. Indeed, the questionnaire had four sections with a total of 95 questions which, added to the detailed PIS and consent form, are burdensome, especially on a hand-held device. Lengthy questionnaires and online surveys are known to receive lower responses[56]. More practicably, a shortened form of the MBI could have been used since abbreviated versions have been found to correlate strongly with their respective domain scores from the full MBI and give identical results in the estimation of associations [9].

### Recommendations

Addressing contextual factors related to organizational arrangements and management systems in the work setting, most importantly workload, leadership, and reward, is key to controlling burnout among RDs and consultants in Nigeria. In this regard, the following measures should be adopted by Nigerian policy makers and health system managers.

1. More emphasis should be placed on the mental health of graduate medical trainees and trainers. There should be an intervention to create more awareness of burnout among RDs and consultants through continuous training programs that incorporate coping strategies for overcoming work stress and preventing burnout. These should include time management, handling difficult clinical cases, satisfying home—work demands, mindfulness techniques, cognitive behavioral therapy, etc. Periodic screening as well as counselling systems should also be instituted[15,29,30,32].
2. Strategies for stress reduction and revision of workload distribution in tertiary hospitals are needed, e.g., a limit on the number of work hours per week, number of overnight calls per month, etc., and stipulating adequate breaks and vacations [15,49,57]. Health systems should be structured to make workload manageable by not placing excessively high demands on RDs, or stifling their autonomy at work [33,58].
3. There is a need for a paradigm shift from the traditional paternalistic form of medical leadership, which is based only on the merits of years of service, academic excellence, and professional competence with little or no emphasis on leadership capacity[59]. With the increasing complexity in medical practice and health systems, medical leadership in Nigeria should also evolve. Appointees to the positions of Chief Medical Director and Director of Clinical Services and Training (at the level of the hospital), Residency Program Coordinator (at the level of the department), and other management positions in healthcare organizations should be persons with vision and well-schooled in critical components of leadership including effective communication, engendering teamwork/supportive organizational culture, and giving adequate recognition to the accomplishments of subordinates [59,60]. “Professional staff not suited to leadership, either through training, selection or natural inclination, should not be entrusted with administrative and management burdens” if possible. [61]. The curricula for medical education, starting from the first professional degree in medicine, should be revised to promote training in medical leadership, prepare future managers, and enshrine the culture of leadership from the early stages of their careers [59]. This can be achieved by incorporating training modules in medical schools, residency training programs, and the methods of continuing medical education so that leadership is taught and reinforced across the continuum of medical training. The modules should cover leadership skills vital in clinical settings, such as communication (including listening), risk assessment, decision-making, empathy, provision of support, confidence, optimism, and transparency[62].
4. Mentorship programs should be incorporated into the residency training in all accredited health facilities, geared at giving younger doctors all the support they can get since they are more prone to burnout [63].
5. Adequate reward systems should be designed. Mechanisms should be instituted for the motivation of medical personnel to stimulate job enthusiasm so that they perform their duties with optimum interest and satisfaction in pursuit of organizational and personal goals[64]. Extrinsic motivation in the form of tangible and intangible incentives from managers of health systems and organizations, such as bonuses, recognitions, and salary increases, can be helpful in controlling burnout among RDs and consultants in Nigeria, especially against the backdrop of the current poor work conditions that encourage constant brain drain[21,65].
6. More research on the issue of burnout among RDs and consultants in Nigeria is recommended. Qualitative studies to give a better understanding of these physicians’ experiences of burnout, and longitudinal studies to identify causal factors will support control efforts.

## Conclusion

This study examined the prevalence of and factors associated with burnout among RDs and consultants in the two tertiary hospitals in Delta State, Nigeria. It showed an overall high burnout of 40.4%, with significant predictors being age, workload, leadership, and reward. It suggests significant levels of burnout among a key component of the medical workforce in Nigeria whose mental health and productivity have grave implications for health care. It also reveals predictors that highlight critical areas for intervention in Nigerian healthcare organizations, especially those involved in residency training. Given the dire consequences of physician burnout on patient care, this study highlights the need for national healthcare policies and interventions that enhance the work life of physicians based on a framework of stress reduction, manageable workloads, effective leadership, and adequate rewards. This should be viewed as fundamental to making the best of health system performance, optimizing patient satisfaction, improving population health, and cutting healthcare costs [18,57,60].

Though this study offers valuable insights into the factors contributing to physician burnout in a Nigerian setting, the findings might also be adapted or applied to address burnout in similar resource-constrained locales of low- and middle-income countries by focusing on the specific contextual factors that could be exacerbating physician burnout in those environments and implementing tailored interventions.

## Supporting information

S1 Data Minimal data set. (SAV)

## References

[1] Kumar S. Burnout and doctors: prevalence, prevention and intervention. Healthcare (Basel). 2016;4(3):37. doi: 10.3390/healthcare4030037 27417625 PMC5041038

[2] Adisa T, Abdulraheem I. “Long working hours and the challenges of work-life balance: the case of Nigerian medical doctors.” In: British Academy of Management (BAM) 2016 Conference Proceedings; 2016 Sep 6-8; Newcastle, United Kingdom. London: British Academy of Management; 2016.

[3] Ilesanmi O. Why Nigeria’s Doctors Are Leaving, And How The Problem Can Be Fixed. The Conversation. 2019 Jul 1 [cited 2021 April 20]. Available from: https://theconversation.com/why-nigerias-doctors-are-leaving-and-how-the-problem-can-be-fixed-117860

[4] World Health Organization. Burn-out An “Occupational Phenomenon”: International Classification of Diseases. 2019 May 28 [cited 2021 May 8]. In: WHO News [Internet]. Geneva: WHO. Available from: https://www.who.int/news/item/28-05-2019-burn-out-an-occupational-phenomenon-international-classification-of-diseases

[5] James T. Making Wellness an Organizational Priority in Health Care. 2021 Apr 23 [cited 2021 May 8] In: Trends in Medicine [Internet]. Boston: Harvard Medical School. Available from: https://postgraduateeducation.hms.harvard.edu/trends-medicine/making-wellness-organizational-priority-health-care

[6] Stehman CR, Testo Z, Gershaw RS, Kellogg AR. Erratum: this article corrects: “burnout, drop out, suicide: physician loss in emergency medicine, Part 1”. West J Emerg Med. 2019;20(5):840–1. doi: 10.5811/westjem.2019.8.44927 31539344 PMC6779705

[7] Shanafelt TD, Balch CM, Bechamps G, Russell T, Dyrbye L, Satele D, et al. Burnout and medical errors among American surgeons. Ann Surg. 2010;251(6):995–1000. doi: 10.1097/SLA.0b013e3181bfdab3 19934755

[8] Wallace JE, Lemaire JB, Ghali WA. Physician wellness: a missing quality indicator. Lancet. 2009;374(9702):1714–21. doi: 10.1016/S0140-6736(09)61424-0 19914516

[9] West CP, Dyrbye LN, Shanafelt TD. Physician burnout: contributors, consequences and solutions. J Intern Med. 2018;283(6):516–29. doi: 10.1111/joim.12752 29505159

[10] Nwosu ADG, Ossai E, Onwuasoigwe O, Ezeigweneme M, Okpamen J. Burnout and presenteeism among healthcare workers in Nigeria: Implications for patient care, occupational health and workforce productivity. J Public Health Res. 2021;10(1):1900. doi: 10.4081/jphr.2021.1900 33634041 PMC7883015

[11] Parsa-Parsi RW. The revised declaration of geneva: a modern-day physician’s pledge. JAMA. 2017;318(20):1971–2. doi: 10.1001/jama.2017.16230 29049507

[12] Ogunsuji O, Adebayo O, Olaopa O, Efuntoye O, Agbogidi J, Kanmodi K, et al. Burnout among Nigerian Doctors: a systematic review. Niger Med Pract. 2019; 76(1-3): 24–29.

[13] Mind Garden Inc. Maslach Burnout ToolkitTM. 2019 [cited 2021 April 30]. In: Mind Garden: Tools For Positive Transformation [Internet]. Menlo Park: Mind Garden, Inc. Available from: https://www.mindgarden.com/184-maslach-burnout-toolkit.

[14] Mind Garden Inc. MBI: Human Services Survey For Medical Personnel. 2019 [cited 2021 October 30]. In: Mind Garden: Tools For Positive Transformation [Internet]. Menlo Park: Mind Garden, Inc. Available from: https://www.mindgarden.com/315-mbi-human-services-survey-medical-personnel.

[15] Ogboghodo EO, Edema OM. Assessment of burnout amongst resident doctors in Benin City, Edo State, Nigeria. Niger Postgrad Med J. 2020;27(3):215–23. doi: 10.4103/npmj.npmj_37_20 32687122

[16] Maslach C, Jackson S, Leiter M. The maslach burnout inventory: third edition. In: Zalaquett C, Wood R, editors. Evaluating stress: a book of resources. Lanham: Scarecrow Press; 1997. pp.191–218.

[17] Legassie J, Zibrowski EM, Goldszmidt MA. Measuring resident well-being: impostorism and burnout syndrome in residency. J Gen Intern Med. 2008;23(7):1090–4. doi: 10.1007/s11606-008-0536-x 18612750 PMC2517942

[18] Mahmood SBZ, Zahid A, Nasir N, Tahir M, Ghouri U, Almas A. Triggering and protective factors of burnout in medical resident physicians in a lower-middle-income country: A cross-sectional study. Ann Med Surg (Lond). 2021;67:102500. doi: 10.1016/j.amsu.2021.102500 34188912 PMC8219648

[19] Thomas NK. Resident burnout. JAMA. 2004;292(23):2880–9. doi: 10.1001/jama.292.23.2880 15598920

[20] Dyrbye LN, West CP, Shanafelt TD. Defining burnout as a dichotomous variable. J Gen Intern Med. 2009;24(3):440; author reply 441. doi: 10.1007/s11606-008-0876-6 19130150 PMC2642563

[21] Anyaehie U, Anyaehie U, Nwadinigwe C, Emegoakor C, Ogbu V. Surgical resident doctor’s perspective of their training in the southeast region of nigeria. Ann Med Health Sci Res. 2012;2(1):19–23. doi: 10.4103/2141-9248.96931 23209985 PMC3507131

[22] National Post-graduate Medical College of Nigeria. Establishment and Functions. 2021 [cited 2021 May 6]. In: About the National Post-graduate Medical College of Nigeria [Internet]. Lagos: National Postgraduate Medical College of Nigeria. Available from: https://npmcn.edu.ng/about-us/establishment-and-functions/.

[23] Awojulugbe O. Explainer: What You Need To Know About Nigeria’s Resident Doctors and Why They Strike So Frequently. 2021 Aug 19 [cited 2022 March 2]. In: Africa Check [Internet]. Johannesburg: Africa Check. Available from: https://africacheck.org/fact-checks/blog/explainer-what-you-need-know-about-nigerias-resident-doctors-and-why-they-strike.

[24] Medical and Dental Consultants Association of Nigeria. Welcome to MDCAN. 2020 [cited 2022 Mar 2]. In: Medical and Dental Consultants Association of Nigeria [Internet]. Ibadan: Medical and Dental Consultants Association of Nigeria. Available from: https://www.mdcan.org.ng/.

[25] Abdelghani M, El-Gohary H, Fouad E, Hassan M. Addressing the relationship between perceived fear of COVID-19 virus infection and emergence of burnout symptoms in a sample of Egyptian physicians during COVID-19 pandemic: a cross-sectional study. Middle East Curr Psychiatry. 2020;27(1):70–78. doi: 10.1186/s43045-020-00079-0.

[26] Soltan M, Soliman S, Al-Hassanin S, ElSherief W, Elnaggar M, Gohar S. Burnout and work stress among medical oncologists: Egyptian multi-centric study. Middle East Curr Psychiatry. 2020;27(1):40–45. doi: 10.1186/s43045-020-00046-9.

[27] Khan FA, Shamim MH, Ali L, Taqi A. Evaluation of job stress and burnout among anesthesiologists working in academic institutions in 2 major cities in Pakistan. Anesth Analg. 2019;128(4):789–95. doi: 10.1213/ANE.0000000000004046 30883424

[28] Jalili M, Sadeghipour Roodsari G, Bassir Nia A. Burnout and associated factors among iranian emergency medicine practitioners. Iran J Public Health. 2013;42(9):1034–42. 26060665 PMC4453883

[29] Ratnakaran B, Prabhakaran A, Karunakaran V. Prevalence of burnout and its correlates among residents in a tertiary medical center in Kerala, India: A cross-sectional study. J Postgrad Med. 2016;62(3):157–61. doi: 10.4103/0022-3859.184274 27320952 PMC4970341

[30] Fernando BMS, Samaranayake DL. Burnout among postgraduate doctors in Colombo: prevalence, associated factors and association with self-reported patient care. BMC Med Educ. 2019;19(1):373. doi: 10.1186/s12909-019-1810-9 31619216 PMC6794729

[31] Nwosu ADG, Ossai EN, Mba UC, Anikwe I, Ewah R, Obande BO, et al. Physician burnout in Nigeria: a multicentre, cross-sectional study. BMC Health Serv Res. 2020;20(1):863. doi: 10.1186/s12913-020-05710-8 32928201 PMC7489005

[32] Omar DI, Hani BM, Abd-Ellatif EE. Burnout among physicians in Egypt during COVID-19 pandemic. Egypt J Hosp Med. 2021;82(4):599–608.

[33] Zeijlemaker C, Moosa S. The prevalence of burnout among registrars in the School of Clinical Medicine at the University of the Witwatersrand, Johannesburg, South Africa. S Afr Med J. 2019;109(9):668–72. doi: 10.7196/SAMJ.2019.v109i9.13667 31635592

[34] Ghazanfar H, Chaudhry MT, Asar ZU, Zahid U. Compassion satisfaction, burnout, and compassion fatigue in cardiac physicians working in tertiary care cardiac hospitals in Pakistan. Cureus. 2018;10(10):e3416. doi: 10.7759/cureus.3416 30542630 PMC6284868

[35] Lrago T, Asefa F, Yitbarek K. Physicians’ burnout and factors in Southern Ethiopia affecting it. Ethiop J Health Sci. 2018;28(5):589–98. doi: 10.4314/ejhs.v28i5.10 30607074 PMC6308775

[36] Yu ITS, Tse SLA. Workshop 6--sources of bias in cross-sectional studies; summary on sources of bias for different study designs. Hong Kong Med J. 2012;18(3):226–7. 22665687

[37] Obong D, Adesoye O, Ochei O, Ntaji M, Okolugbo N, Owa-Nwabuzo O. The index case of covid-19 in Delta State, Nigeria: a case report. J Com Med and PHC. 2022;34(1):58–68. doi: 10.4314/jcmphc.v34i1.4

[38] Joshua BW, Fuwape I, Rabiu B, Pires EES, Sa’id RS, Ogunro TT, et al. The impact of the first and second waves of COVID-19 pandemic in Nigeria. Geohealth. 2023;7(3):e2022GH000722. doi: 10.1029/2022GH000722 36968154 PMC10030272

[39] Busari SI, Samson TK. Modelling and forecasting new cases of Covid-19 in Nigeria: Comparison of regression, ARIMA and machine learning models. Sci Afr. 2022;18:e01404. doi: 10.1016/j.sciaf.2022.e01404 36310608 PMC9595487

[40] Johns Hopkins University and Medicine. Nigeria: Overview. 2023 Mar 10 [cited 2025 February 7]. In: Corovirus Resource Center [Internet]. Baltimore: Johns Hopkins University and Medicine. Available from: https://coronavirus.jhu.edu/region/nigeria.

[41] Fatunmole M. COVID-19: why Nigeria slipped into fourth wave. 2021 Dec 21 [cited 2025 February 7]. In: International Centre for Investigative Reporting [Internet]. Lagos: International Centre for Investigative Reporting. Available from: https://www.icirnigeria.org/covid-19-why-nigeria-slipped-into-fourth-wave/.

[42] Agi V. Lagos says COVID-19 4th wave over, as WHO reports cases decline in Africa. Prime Progress. 2023 Dec 13 [cited 2025 February 7]. Available from: https://primeprogressng.com/perspective/lagos-says-covid-19-4th-wave-over-as-who-reports-cases-decline-in-africa/.

[43] Oluwafemi A. NCDC: Nigeria now in fourth wave of COVID. . .cases increased by 500% in two weeks. The Cable. 2021 Dec 20 [cited 2025 February 7]. Available from: https://www.thecable.ng/breaking-nigeria-now-in-fourth-wave-of-covid-says-ncdc/.

[44] Ndiaye AJS, Beye M, Sow A, Lo G, Padane A, Sokhna C, et al. COVID-19 in 16 West African countries: an assessment of the epidemiology and genetic diversity of SARS-CoV-2 after four epidemic waves. Am J Trop Med Hyg. 2023;109(4):861–73. doi: 10.4269/ajtmh.22-0469 37640294 PMC10551082

[45] Tolu-Kolawole D. Nigeria now in COVID-19 fourth wave, says NCDC. Punch Healthwise. 2021 Dec 21 [cited 2025 February 7]. Available from: https://healthwise.punchng.com/nigeria-now-in-covid-19-fourth-wave-says-ncdc/.

[46] Mind Garden Inc. Transform Survey Hosting (Data): Toolkit for Medical Personnel. 2019 [cited 2021 Jul 15]. In: Mind Garden: Tools for Positive Transformation [Internet]. Menlo Park: Mind Garden, Inc. Available from: https://www.mindgarden.com/maslach-burnout-toolkit-for-medical-personnel/695-awsmbi-hssmp-transform-survey-hosting.html.

[47] Mind Garden Inc. Maslach Burnout ToolkitTM for Medical Personnel. 2019 [cited 2021 Apr 30]. In: Mind Garden: Tools for Positive Transformation [Internet]. Menlo Park: Mind Garden, Inc. Available from: https://www.mindgarden.com/329-maslach-burnout-toolkit-for-medical-personnel.

[48] Mind Garden Inc. Pandemic Experiences & Perception Survey. 2022 [cited 2022 Jan 10]. In: Mind Garden: Tools for Positive Transformation [Internet]. Menlo Park: Mind Garden, Inc. Available from: https://www.mindgarden.com/346-pandemic-experiences-perceptions-survey#horizontalTab4.

[49] Goyal P, Rustagi N, Belkić K. Physicians’ total burden of occupational stressors: more than threefold increased odds of burnout. South Med J. 2021;114(7):409–15. doi: 10.14423/SMJ.0000000000001277 34215893

[50] Taiwo K. More female doctors, less on duty. Dataphyte. 2024 Aug 24 [cited 2025 February 8]. Available from: https://dataphyte.com/latest-reports/more-female-doctors-less-on-duty/

[51] Churm P. Doctors in Nigeria continue strike action over pay and conditions. Africanews. 2024 Aug 13 [cited 2025 February 2]. Available from: https://www.africanews.com/2023/07/28/doctors-in-nigeria-continue-strike-action-over-pay-and-conditions/

[52] Nigeria Health Watch. Healthcare Leadership in Nigeria needs a “radical shift”. 2016 Nov 9 [cited 2025 February 5]. In: Nigeria Health Watch [Internet]. Lagos: Nigeria Health Watch. Available from: https://articles.nigeriahealthwatch.com/healthcare-leadership-in-nigeria-needs-a-radical-shift/.

[53] Ranganathan P, Pramesh CS, Aggarwal R. Common pitfalls in statistical analysis: Logistic regression. Perspect Clin Res. 2017;8(3):148–51. doi: 10.4103/picr.PICR_87_17 28828311 PMC5543767

[54] Hulley S, Cummings S, Browner W, Grady D, Newman T. Designing clinical research. 4th ed. Philadelphia: Lippincott Williams and Wilkins; 2013.

[55] Bruce N, Pope D, Stanistreet D. Quantitative methods for health research: A practical interactive guide to epidemiology and statistics. 2nd ed. Chichester: John Wiley and Sons; 2008.

[56] Bowling A. Research methods in health: investigating health and health Services. 4th ed. Berkshire: Open University Press; 2014.

[57] NIkenna NK, Ewenitie E-UF, Chidiebere ODI, Uchenna E, Isaac AN, Ezinne NI. Evaluation of physician burnout syndrome among pediatric resident doctors in Nigeria. Nigerian J Med. 2021;30(2):182–6. doi: 10.4103/njm.njm_134_20

[58] Dyrbye L, Shanafelt T. A narrative review on burnout experienced by medical students and residents. Med Educ. 2016;50(1):132–49. doi: 10.1111/medu.12927 26695473

[59] Loughlin KR. Medical Leadership: Past, Present and Future. 2022 Jan 26 [cited 2022 March 9]. In: Trends in Medicine [Internet]. Boston: Harvard Medical School. Available from: https://postgraduateeducation.hms.harvard.edu/trends-medicine/medical-leadership-past-present-future.

[60] Nmadu A, Omole V, Oyefabi A, Usman N, Igboanusi C, Gobir A, et al. Physician burnout: An overview. J Res Basic Clin Sci. 2019;1(1):111–114.

[61] Park K. Park’s textbook of preventive and social medicine. 22nd ed. Jabalpur, India: M/s Banarsidas Bhanot; 2013.

[62] Isibor E, Kanmodi K, Adebayo O, Olaopa O, Igbokwe M, Adufe I, et al. Exploring issues and challenges of leadership among early career doctors in nigeria using a mixed-method approach: CHARTING study. Eur J Investig Health Psychol Educ. 2020;10(1):441–54. doi: 10.3390/ejihpe10010033 34542496 PMC8314232

[63] Shanafelt TD, Sloan JA, Habermann TM. The well-being of physicians. Am J Med. 2003;114(6):513–9. doi: 10.1016/s0002-9343(03)00117-7 12727590

[64] Olise P. Fundamentals of leadership and management in primary health care. Abuja: Ozege Publications; 2011.

[65] Szigethy E. “Burnout”: strategies to prevent and overcome a common—and dangerous—problem. Psychiatric Times. 2014 May 29 [Cited 2025 February 7]. Available from: https://www.psychiatrictimes.com/view/burnout-strategies-prevent-and-overcome-commonand-dangerousproblem.

