# Supplementary material for: Physician burnout in the context of the COVID-19 pandemic: prevalence and associated factors among resident doctors and consultants in Delta State, Nigeria
Source: PLOS Ment Health. 2025 May 7;2(5):e0000175. doi: 10.1371/journal.pmen.0000175 (PMC12798376; doi:10.1371/journal.pmen.0000175)
Supplement: Attachment — Submitted filename: Response to Reviewers.docx [file pmen.0000175.s003.docx]

**RESPONSE TO REVIEWERS**

**(***The points raised by the editor and reviewers are in blue italics.*

The responses of the authors are in black**)**

A. ***Editor Comments***
1. *When completing the data availability statement of the submission form, you indicated that you would make your data available on acceptance. We strongly recommend all authors decide on a data-sharing plan before acceptance, as the process can be lengthy and hold up publication timelines. Please note that, though access restrictions are acceptable now, your entire data will need to be made freely accessible if your manuscript is accepted for publication. This policy applies to all data except where public deposition would breach compliance with the protocol approved by your research ethics board. If you are unable to adhere to our open data policy, please kindly revise your statement to explain your reasoning and we will seek the editor's input on an exemption. Please be assured that, once you have provided your new statement, the assessment of your exemption will not hold up the peer review process.*

We have made our data available with the submission of the revised manuscript

2. *We do not publish any copyright or trademark symbols that usually accompany proprietary names, eg (R), (C), or TM  (e.g. next to drug or reagent names). Please remove all instances of trademark/copyright symbols throughout the text, including “Transform™ Survey Hosting”, “Maslach Burnout Toolkit™”, and “Maslach Burnout Inventory™” on page 14.*

All trademark/copyright symbols have been removed

B. ***Reviewer #1 Comments***

***Abstract***

1. *The first sentence could be clearer. Instead of "Residents Doctors (RDs) and consultants carry out the most specialized medical care," it may be more accurate to state: "Resident Doctors (RDs) and consultants provide specialized medical care." Also, consider introducing the term "burnout" earlier in the background to ensure it is immediately clear why the study is relevant.*

The suggested edits have been effected.

2. *The phrase "Previously validated instruments were used to collect data via an online survey" could benefit from mentioning the specific instruments used (e.g., Maslach Burnout Inventory). This would provide a more concrete understanding of the methodology.*

Thank you for the suggestion. We have incorporated the specific instrument in the sentence.

3. *The conclusion is strong, but it might be helpful to explicitly state the significance of the findings for future interventions in Nigerian healthcare systems.*

Done

***Introduction***

1. *In the first paragraph, the definitions of "resident doctor" and "consultant" are useful but could be more concise. The current phrasing is somewhat repetitive with the following sentences. Try simplifying for brevity.*

The somewhat repetitive sentence has been deleted.

2. *The burnout definition is clear, but it might be beneficial to emphasize its relevance to healthcare professionals earlier in the introduction. The mention of “incessant brain drain” and “unconducive conditions” is important, but the connection to burnout could be made more explicit here rather than later in the text*

The relevant adjustments have been made

3. *Consider explaining briefly how the COVID-19 pandemic has exacerbated the challenges faced by physicians in the country. For example, the strain on healthcare systems due to COVID-19 could be mentioned in the context of these pre-existing issues.*

Done

4. *While it is crucial to emphasize the role of work-related stressors, consider streamlining the points about “lack of essential tools/equipment, inefficient systems, poor remuneration, and absence of support” to a more concise list. Perhaps combining these under broader categories like “resource challenges” and organizational challenges” could help improve flow.*

Done

5. *The introduction does well to highlight the gap in research regarding physician burnout in low- and middle-income countries, especially during the COVID-19 pandemic. The comparison with other regions could be strengthened by mentioning some specific studies or data from similar contexts to establish a stronger foundation for the study's significance. For example* <https://www.frontiersin.org/journals/psychiatry/articles/10.3389/fpsyt.2024.1373743/full> , <https://journals.plos.org/plosone/article?id=10.1371/journal.pone.0309701> , <https://bmjopen.bmj.com/content/11/9/e054284.abstract> , <https://journals.plos.org/plosone/article?id=10.1371/journal.pone.0305713>

Thank you for the suggestion. We already referred to all the studies in the literature at the time of writing that used the Maslach Burnout Inventory (MBI), which is the gold standard, in their measurement of burnout. The suggested studies used the Professional Quality of Life Scale (ProQOL-5).

6. *How do the research objectives build upon previous studies in Nigeria, and how does this study address gaps identified in the literature?*

As we mentioned toward the end of the introduction, there is a paucity of studies in Nigeria involving both resident doctors and consultants, assessing organizational factors, and providing data on physician burnout in the context of COVID-19. The need to fill these gaps informed the objectives of this study

7. *Could you elaborate on the rationale for focusing on tertiary hospitals in Delta State, Nigeria?*

As we stated while defining Resident Doctors (RDs) in the introduction, tertiary hospitals have by far the highest concentrations of RDs and consultants in any medical specialty.

**Methods**

1. *It would be helpful to briefly mention why a cross-sectional design was chosen, particularly in relation to the study objectives.*

Done

2. *Consider providing additional information on the characteristics of the facilities, such as the specific types of services they provide or their size. This could provide a better context for understanding the generalizability of the findings.*

Done

3. *It might be helpful to mention whether the study includes any specific characteristics or exclusions within the group of RDs and consultants (e.g., specialty, gender, etc.), if applicable*

We mentioned, under the Selection Criteria section, that only RDs and consultants who had worked in the facilities for at least one year were included. Also, supernumerary RDs and consultants hired on temporary or part-time contracts were excluded.

4. *In the multistage sampling technique, you might want to clarify how the participants were selected within the specialty and cadre groups*

Thanks for pointing this out. We have added that “Participants were then selected by simple random sampling technique (balloting method) for individual cadres in each specialty for the two hospitals.”

5. *Could you clarify why the specific instruments and scales used (e.g., MBI) were chosen over other potential tools.*

We already mentioned in the introduction that the Maslach Burnout Inventory (MBI) is the gold standard and the most prominent tool for measuring burnout as defined by the World Health Organization. It has been validated by countless research on burnout that have been done since the tool was developed over four decades ago. Furthermore, we added in the ‘Method of Data Collection’ that the Areas of Worklife Survey (AWS), a companion tool to the MBI that assesses workplace characteristics that could predict burnout in the work-life context, has proven validity and reliability in different occupational settings. We also stated that the PEPS is a powerful tool for measuring employees’ experiences during a pandemic.

6. *Was there any pretesting of the survey instrument to ensure suitability for the study population?*

Yes, the survey instruments were pretested among 11 RDs and 10 consultants of a teaching hospital in a neighboring state to ensure suitability for the study population, and they were revised for clarity. This has been added to the manuscript.

7. *Given the low response rate, did you perform any sensitivity analysis or adjustments to account for potential response bias?*

No, we did not perform any sensitivity analysis or adjustments. The implications of the low response rate were acknowledged in our discussion of the study’s limitations. We stated that the extent of the non-response bias could have been determined by comparing the characteristics of responders with the same attributes of non-responders, but tangible information about the population was unavailable.

8. *It might be useful to briefly explain how the PEPS was adapted or validated for this particular study.*

The PEPS had to be adapted for this study by slightly modifying the questions to make them specific to the current pandemic. For example, in the section on ‘Leadership’, the question “How did you experience leadership during the pandemic period” was changed to “How do you experience leadership during the ongoing COVID-19 pandemic.” Similarly, in the section on ‘Risk Perception’, “For the ongoing COVID-19 pandemic, how much risk to your family do you perceive” was used instead of “For the relevant period, how much risk to your family did you perceive.”

9. *The authors should include information on how the online format might have impacted participation or data quality (e.g., completion rates, dropout rates, etc.)*

We already stated in the discussion of the study’s limitations that online surveys and lengthy questionnaires – our questionnaire had 95 questions in total - are known to receive lower responses.

10. *The authors should mention any steps taken to check for multicollinearity or other issues that could affect the validity of the regression model*

Unfortunately, no such steps were taken.

11. *Consider briefly mentioning any steps that were taken to protect participant confidentiality and anonymity beyond password protection (e.g., data storage procedures, who had access to the data, etc.)*

We had stated that there were no identifiers on the questionnaire, and the online survey did not have any IP links, so it remained anonymous. We have now added that the data was stored in a password-protected laptop that was accessible to the investigators only.

12. *Clarify if there were any incentives for participation or compensation for participants*

We have added that there was no incentive or compensation for participants

**Results**

1. *The multivariate analysis presents interesting findings, particularly with the likelihood of older age groups experiencing lower EE. However, the authors should clarify what other variables were controlled for in this analysis to strengthen the interpretation of the results. For instance, did the analysis control for years of experience or professional rank when assessing the effects of age on burnout?*

As we stated in the section on Data Analyses as regards the plan for identifying independent predictors of each domain of burnout, only the categorical and continuous variables that were significant on bivariate analyses (Chi-squared test and Pearson correlation) were introduced into the binary logistic regression model. ‘Professional Rank’ was not statistically significant on the Chi-squared test for association with EE, so there was no need to control for it. ‘Age’ and ‘Years of experience’ (‘Number of years post-graduation’) were statistically significant on the Chi-squared test, and scores for work hours per week, workload, control, reward, community, fairness, values, and adequacy of resources were significant on Pearson correlation. These were included in the multivariate analysis; and only age, workload, and reward turned out to be independent predictors of EE.

***Discussion***

1. *You mention that the burnout levels in your study are lower than in certain other African countries. Could you clarify the key contextual differences that might explain this variance?*

We already stated, shortly after noting this observation, that the higher rates in those studies could be because those studies involved only resident doctors (who are younger doctors and undergo more work stress) or were focused on one specialty with a greater tendency for burnout such as emergency medicine.

2. *How do the observed associations between burnout and contextual factors such as workload and leadership contribute to the broader understanding of physician burnout in resource-constrained settings?*

Thanks for drawing our attention to this. We have added that “The observed association between burnout and contextual factors such as workload and leadership contributes to a broader understanding of physician burnout in resource-constrained settings by highlighting how these elements interact to create an environment where burnout is more likely to occur and more difficult to mitigate. In Nigeria, physicians continue to face high patient volumes, insufficient staffing, limited tools, and poor remuneration. It is also acknowledged that healthcare professionals occupying leadership positions in both public and private sectors often lack the needed leadership skills and competencies. Recognizing these patterns can lead to more effective strategies aimed at supporting physician well-being and improving patient care in these challenging settings.

3. *In the case of gender and burnout, you note no significant association. Could you provide more insight into potential cultural or systemic factors that might explain this finding in your context?*

Thanks for pointing this out! We have highlighted some considerations. The unique cultural and systemic factors in Nigeria and similar settings could explain why gender does not emerge as a significant predictor of burnout. Generally, male and female physicians face enormous societal expectations, although these might differ in form. For example, while women may be expected to juggle professional and domestic responsibilities (given traditional gender roles around caregiving and homemaking), men may experience the pressure of being the primary earners and decision-makers of their families. However, the distinction in these forms of stress may not be significant enough to differentiate the burnout experiences between the two genders, especially if both groups encounter similar systemic challenges such as high workloads and lack of resources in their professional life. Secondly, though the medical profession in Nigeria continues to be male-dominated, the expectations for physicians are the same, regardless of gender. Female physicians, particularly those entering the field in recent decades, are usually expected to adapt to their demanding roles just like their male counterparts.

***Limitations/Strengths***

1. *Regarding the sample size and low response rate, do you think the burnout levels reported might underestimate the true prevalence? How might this impact the generalizability of your findings?*

We already stated in the study limitations that the low response rate has implications for external validity since the sample may be unrepresentative of the study population, as individuals who turn down research invitations tend to differ from those who participate. We also noted that the low sample size and reduced statistical power meant that the survey estimates might not be precise. Furthermore, we mentioned that one potential participant contacted the researchers to let us know that he started the survey but could not complete it because the questions triggered him. This suggests that the obtained prevalence of burnout is less than the true picture since non-response, even as low as 25%, distorts estimates of disease burden when the disorder itself is responsible for the non-response.

2. *Could you provide more detail on how you addressed emotional discomfort among participants, particularly in cases where distress might be linked to burnout itself?*

We already stated that, as a duty of care, the physician who let us know that he experienced emotional distress was directed to organizations where he could get help if he thought it necessary. Furthermore, we mentioned in the ‘Ethical Considerations’ that the Participant Information Sheet (PIS) contained contact details of support groups/organizations that participants could reach if the questionnaire caused emotional distress. The PIS also had the researchers’ telephone numbers and email addresses to enable potential participants to ask questions if needed.

***Recommendations***

1. *The recommendations emphasize improving leadership capacity. Did your findings identify specific gaps in current leadership training or practices that need to be addressed?*

The objective of the study was not to assess current leadership training or practices per se. However, in keeping with our aim, we observed that the scores for burnout (Emotional Exhaustion and Depersonalization) among physicians had a statistically significant negative correlation with scores for experience of good leadership. As we have noted in the discourse on how the observed association between burnout and contextual factors contributes to the broader understanding of physician burnout in resource-constrained settings, it is acknowledged that healthcare professionals occupying leadership positions in both the public and private sectors in Nigeria often lack the needed leadership skills and competencies. Recognizing these patterns can lead to more effective strategies that support physician well-being and improve patient care in these challenging settings.

2. *You propose a revision of medical education curricula to include leadership training. Could you clarify how you envision this being implemented within the Nigerian medical education system?*

Thank you for this suggestion! We have added that this can be achieved by incorporating training modules in medical schools, residency training programs, and the methods of continuing medical education so that leadership is taught and reinforced across the continuum of medical training. The modules should cover leadership skills vital in clinical settings, such as communication (including listening), risk assessment, decision-making, empathy, provision of support, confidence, optimism, and transparency.

***Conclusion***

1. *Could you expand on how your findings might influence national healthcare policies, particularly the brain-drain issue and its connection to physician burnout?*

We have added that this study highlights the need for national healthcare policies and systems that enhance the work life of physicians based on a framework of stress reduction, manageable workloads, effective leadership, and adequate rewards.

2. *How might your study’s findings be adapted or applied to address burnout in other regions of Nigeria or similar low- and middle-income countries?*

We have added that “Though this study offers valuable insights into the factors contributing to physician burnout in a Nigerian setting, the findings might also be adapted or applied to address burnout in similar resource-constrained locales of low- and middle-income countries by focusing on the specific contextual factors that could be exacerbating physician burnout in those environments and implementing tailored interventions.”

C. ***Reviewer 2 Comments***

*Data was collected between 13^th^ January and 5^th^ March 2022. Please add when the pandemic began in Nigeria, when it peaked and when it waned. This is important as our experience in India was that during the first wave in 2020-21 in India there was widespread psychological distress but by the end of 2021 and in 2022 the psychological distress had waned. In addition, mention the average patient load with COVID-19 in the tertiary care institute.*

Unfortunately, we do not have data on the average patient load with COVID-19 at the time of the study. We added, “The index case of COVID-19 in Nigeria was reported on February 27, 2020, while the first case in Delta State was confirmed on April 7, 2020. The first wave of the pandemic in the country lasted from February to October 2020; the second wave, from October 2020 to June 2021, and the third wave, from June to November 2021. The fourth wave began in December 2021 and flattened in January 2022. The second wave was the largest, with the number of new cases per month reaching a record high of 43,635 in January 2021. Though the fourth wave had the shortest duration, it was also remarkable as new cases increased by 500% in two weeks, and the week of December 20 – 26 witnessed an unprecedented 14,078 new cases per week.
